# Damage Localization and Severity Assessment in Composite Structures Using Deep Learning Based on Lamb Waves

**DOI:** 10.3390/s24248057

**Published:** 2024-12-17

**Authors:** Muhammad Muzammil Azad, Olivier Munyaneza, Jaehyun Jung, Jung Woo Sohn, Jang-Woo Han, Heung Soo Kim

**Affiliations:** 1 Department of Mechanical, Robotics and Energy Engineering, Dongguk University-Seoul, Seoul 04620, Republic of Korea; muzammilazad@dgu.ac.kr (M.M.A.); 2024120250@dgu.ac.kr (J.J.); 2 Department of Aeronautics, Mechanical and Electronic Convergence Engineering, Kumoh National Institute of Technology, Gumi 39177, Republic of Korea; olivis@kumoh.ac.kr; 3 School of Mechanical System Engineering, Kumoh National Institute of Technology, Gumi 39177, Republic of Korea; jwsohn@kumoh.ac.kr (J.W.S.); uddan@kumoh.ac.kr (J.-W.H.)

**Keywords:** deep learning, damage detection, damage localization, severity assessment, convolutional neural network, Lamb wave

## Abstract

In composite structures, the precise identification and localization of damage is necessary to preserve structural integrity in applications across such fields as aeronautical, civil, and mechanical engineering. This study presents a deep learning (DL)-assisted framework for simultaneous damage localization and severity assessment in composite structures using Lamb waves (LWs). Previous studies have often focused on either damage detection or localization in composite structures. In contrast, this study aims to perform damage detection, severity assessment, and localization using independent DL models. Three DL models, namely the artificial neural network (ANN), convolutional neural network (CNN), and gated recurrent unit (GRU), are compared. To assess their damage detection and localization capabilities. Moreover, zero-mean Gaussian noise is introduced as a data augmentation technique to address the variability and noise inherent in LW signals, improving the generalization capability of the DL models. The proposed framework is validated on a composite plate with four piezoelectric transducers, one at each corner, and achieves high accuracy in both damage localization and severity assessment, offering an effective solution for real-time structural health monitoring. This dual-function approach provides a scalable data-driven method to evaluate composite structures, with applications in predictive maintenance and reliability assurance in critical engineering systems.

## 1. Introduction

Among a variety of composite materials, laminated composites stand out, due to their high strength and exceptional mechanical performance. These materials offer significant weight savings, which contribute to their widespread acceptance across various sectors including the marine, automotive, aerospace, and civil engineering industries [1,2]. Under in-service loadings, many interrelated damage mechanisms are sequentially induced in laminated composites, and can potentially lead to catastrophic structural collapse [3]. Thus, ultrasonic guided wave (UGW)-based structural health monitoring (SHM) technology has been created to rapidly detect internal structural abnormalities [4,5,6]. When Lamb waves (LWs), a type of UGW, traverse the flat laminated composite, they are sensitive to local flaws and lose energy during transmission. Generally, piezoelectric (PZT) sensors are attached to the composite structures to detect LWs and convert impulses into electrical signals, to evaluate the location or area of the damage. However, the inherent dispersion features and multimodal occurrences of LWs make it more difficult to extract information related to different damage severities [7]. Examination of LW signals reveals that the current detection methods consist mainly of either physics-based approaches or data-driven approaches.

In the conventional physics-based methods for damage diagnosis and localization in laminated composites, most research focuses on extracting characteristics from the raw ultrasonic-guided waves, which are responsive to common failure types. The LW data is monitored across the entire life of the composites, where the time of flight (TOF) [8], variations in the power spectral density (PSD) [9], or energy scattering (ES) [10], serve as indicators of damage detection in composite structures. According to the physics-based explanation, when LWs propagate through the composite structure, the damage inside causes discontinuities that increase reflection and energy dissipation in the sensor signals. The temporal waveform and spectral energy distribution of LWs are substantially altered by the reflection components brought about by the damage. Thus, to create the physics-based damage detection model, certain damage-sensitive characteristics are chosen as the damage index (DI). To predict transverse matrix fractures in laminated composite structures, Wilson and Chang [9] developed a physical model using PSD and evaluated the DI by identifying the change in the PSD. Muralidhar et al. [11] employed three factors to define damage in the hybrid fiber metal composite laminates. These factors included Young’s modulus, damage magnitude, and damage position throughout the length of the composite, and an inverse Bayesian technique was applied to identify damage. Various other DI-based approaches have utilized the amplitude of the symmetric/antisymmetric (S_0_/A_0_) waves [12], local correlation coefficient [13], and first-to-residual energy ratio [14]. While these physical models are capable of linking the variations in LW signals to the damage in composite structures, it is essential to recognize that, when adapting the models to particular experimental conditions, some degree of approximation is often required. Due to this constraint, the physical model may not apply to a wider range of applications [15]. Moreover, with the rise in complexity of the structural geometry and the circumstances of service environments, as well as when there is a change in the structural features of laminated composites, it is anticipated that the effectiveness of the physics-based model will decrease. In real-world scenarios, this decrease may result in difficulties.

Conversely, the data-driven approaches utilize artificial intelligence (AI) techniques, like machine learning (ML) and deep learning (DL), that seek to derive intricate representations from extensive monitoring data, therefore training a classifier to estimate damage conditions, or a regressor to determine the damage location [16,17]. Among such approaches, Wu et al. [18] used the continuous wavelet transform (CWT) to convert raw LW signals into time–frequency domain images known as scalograms; following this, a 2D-convolutional neural network (2DCNN) model was utilized to determine the state of the composite structure. Lee et al. [19] employed pre-processed UGW signals to train an autoencoder-based DL model to identify fatigue damage in laminated composites, enabling the autonomous extraction of damage-sensitive features from the autoencoder’s feature space. Various other data-driven methods include artificial neural networks [20,21], the Bayesian model [22,23], clustering algorithms [24], the differential evolution algorithm [25], extreme learning machine [26], the Gaussian process regression model [27], the gated recurrent unit [28], the Kalman filter [29], random forest [30], stacked autoencoder [31], the support vector machine [32], and transfer learning [33]. These data-driven approaches are further divided into shallow machine learning models or deep learning models. While shallow learning models can perform well, their effectiveness relies heavily on manually extracted features, which is time-consuming and requires significant domain expertise [34]. Furthermore, different objectives, such as damage detection, severity assessment, and localization, demand separate feature engineering efforts. In contrast, deep learning models autonomously extract task-specific features directly from raw data, enabling adaptability to multiple objectives. Therefore, the deep learning-based data-driven approaches have the potential to match, or even surpass, the performance of physics-based methods, all while requiring minimal domain-specific expertise. However, the limited availability of monitoring data in damaged conditions could lead to the overfitting of data-driven models, ultimately compromising their accuracy in detecting and localizing damage. Thus, their reliability remains a subject of interest, particularly in scenarios with limited amounts of data.

To address the limitations of current methods in damage detection, severity assessment, and localization for laminated composites, a DL-based framework is proposed that directly utilizes raw LW signals, rather than relying on imaging methods. This approach preserves signal fidelity and streamlines the detection process, offering a direct analysis pathway that bypasses the need for intricate signal transformations. Firstly, independent DL models, including artificial neural network (ANN), convolutional neural network (CNN), and gated recurrent units (GRU), are trained to perform both damage severity assessment and localization tasks, thus enabling a comprehensive assessment of structural health. This dual functionality moves beyond the singular focus of either detection or localization seen in many existing studies. Secondly, the DL models are designed to address the challenges of low signal-to-noise ratios in raw LW data by incorporating zero-mean Gaussian noise augmentation. This approach enables the models to autonomously extract damage-related features, eliminating the need for the pre-defined features that are typically required in conventional machine learning methods. The capacity of the model to identify intricate patterns directly from data improves robustness and reduces dependence on the manually extracted hand-crafted features that are required for traditional machine learning approaches. Finally, the proposed approach is validated on a composite plate equipped with piezoelectric (PZT) sensors, which provide LW data for three damage severity levels and nine distinct damage locations.

## 2. Methods

**Figure 1:**
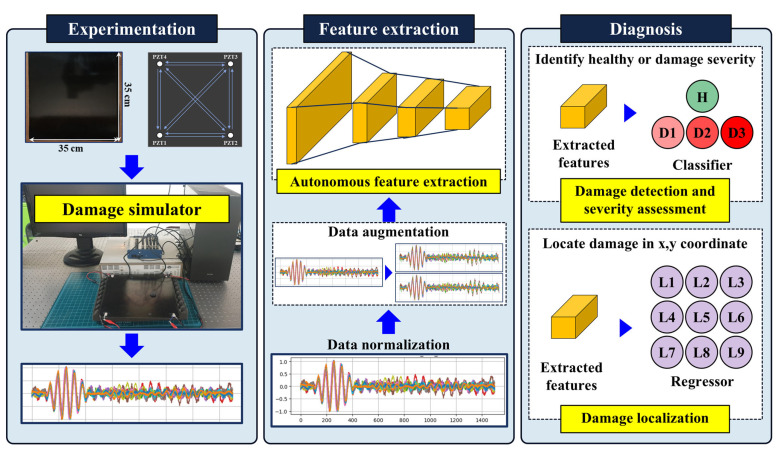
Schematics of the proposed approach, comprising three modules.

Figure 1 illustrates the proposed damage detection and localization framework for laminated composite, and is comprised of three modules: experimentation, feature extraction, and damage diagnosis. In the experimentation module, composite samples are fabricated and subjected to LW experiments to simulate damage conditions that affect the stiffness of the structure due to varying severity levels. This setup enables precise control over damage variables, allowing for data collection across multiple damage severities and locations, which are critical for subsequent analysis. In the feature extraction module, the acquired LW data undergoes a series of pre-processing steps to enhance feature extraction processes. First, data normalization is applied to ensure consistency across the dataset. Then, data augmentation is performed using zero-mean Gaussian noise, generalizing the model by mimicking the variability inherent in real-world conditions. These pre-processed signals are then fed into DL models for autonomous feature extraction. By relying on data-driven methods, rather than manual feature engineering, the framework achieves an end-to-end scalable approach to learning damage-sensitive features directly from raw LW signals, which reduces dependence on expert knowledge and prior assumptions. Finally, the diagnosis module is structured to perform damage detection, severity assessment, and localization. The high-dimensional feature representations extracted in the previous module are utilized in this module to classify the health state of the composite and identify the severity level. Additionally, the model independently localizes the damage using spatial information derived from the sensor layout. This multi-task approach enables comprehensive monitoring, providing an efficient and adaptable method for the real-time SHM of composite structures.

### 2.1. Experimentation

#### 2.1.1. Composite Fabrication

Carbon fiber-reinforced polymer (CFRP) composites are among the most widely used laminated composites; hence, CFRP laminates were fabricated for LW-based SHM experiments using T700SC–12k–60E epoxy-based carbon fiber prepreg. The laminates were manufactured through a hot press compression molding process to ensure uniform thickness and a high-quality fiber-matrix interface with minimal void content. This process is critical, as a weak fiber-matrix interface can act as an artificial delamination in laminated composites, causing unintended wave scattering and reducing the reliability of damage detection. Uniform thickness is also essential as it ensures consistent Lamb wave propagation by maintaining a stable wave velocity and avoiding unwanted reflections or scattering caused by thickness variations. Moreover, square-shaped CFRP laminates were prepared to facilitate the fabrication process, ensuring uniform compression during molding and simplifying the trimming of rough edges before LW testing. The layup sequence consisted of eight plies in a [0/90/0/90]s cross-ply orientation, designed to impart the necessary anisotropic properties to effectively capture LW interactions with internal defects. After the initial fabrication of a 35 cm × 35 cm CFRP sheet, the specimen was trimmed to a 30 cm square to remove rough edges, creating a uniform boundary that was conducive to accurate signal acquisition. This careful specimen preparation facilitates controlled testing across varying damage severities and locations, supporting robust data collection for subsequent DL analysis.

#### 2.1.2. Lamb Wave Testing

**Figure 2:**
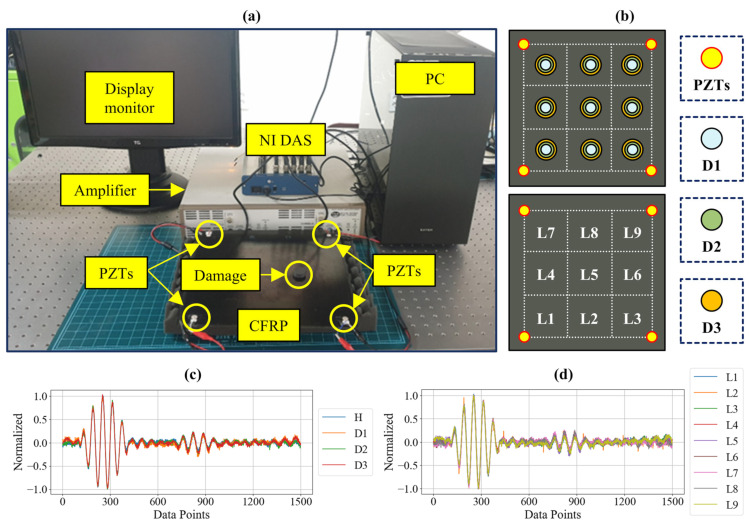
(**a**) The components of the experimental setup, (**b**) schematic representation of CFRP laminated with three damage severities at nine zones, (**c**) the waveform for different damage severities, and (**d**) the waveform obtained for same damage at nine different locations.

To conduct Lamb wave-based testing, a piezoelectric (PZT) sensor array was deployed, with four PZT sensors (PI Ceramic) positioned at each corner of the square CFRP laminate. Such configurations of the PZTs were used to ensure maximum coverage of the area for wave propagation, thus facilitating damage localization. An NI USB−6341 data acquisition system (DAS) was used to excite the PZT sensors and record the response signals. Lamb wave generation and data acquisition were managed using a LabVIEW interface on a connected PC through MATLAB R2022b, which enabled precise control over signal parameters and data flow. A PZD700A dual-channel amplifier was employed to amplify the excitation signal. The experimental setup and components are shown in Figure 2. Each test used a 5-cycle sinusoidal tone burst signal at 150 kHz as the excitation signal. The excitation frequency of 150 kHz was chosen as it falls within the optimal range for generating distinct Lamb wave modes in the CFRP laminates. The reason is that using lower frequencies, such as 100 kHz, results in reduced spatial resolution due to longer wavelengths, which limits sensitivity to small defects, whereas higher frequencies such as 200 kHz result in excessive wave dispersion and energy loss which restricts reliable defect characterization over longer distances [35,36]. In each testing sequence, one of the four PZT sensors was designated as the actuator to generate Lamb waves, while the remaining three PZT sensors served as receivers to capture the propagated wave signals. This configuration allowed for a sequential arrangement where each PZT sensor took turns acting as the actuator, resulting in four separate excitation cycles. During each cycle, the actuator PZT emitted Lamb waves across the CFRP laminate, and the waves were received by the other three PZTs positioned at different corners. By rotating the role of the actuator among the four PZT sensors, a comprehensive network of 12 unique sensing paths was created across the laminate. These sensing paths provide diverse spatial perspectives on Lamb wave propagation, which is critical to accurately identify and localize damage within the composite structure. The DAS collected response signals from each PZT sensor at a sampling frequency of 500 kHz, providing high-resolution data DL models. To simulate damage of varying severities, masses of (6, 12, and 18) g were applied to the laminate, simulating three distinct damage levels (D1, D2, and D3), as shown in Figure 2. This method was chosen for its simplicity, repeatability, and ability to control multiple damage severities [37]. The additional mass changes the local stiffness of the laminate, altering Lamb wave propagation characteristics such as amplitude and phase, enabling effective damage simulation without artificially induced delamination. Moreover, testing with artificially induced delaminations would require multiple laminates with delaminations at various locations introducing challenges during and lower precision in locating the delaminations as they are not visible externally. For localization, the CFRP sheet was divided into nine zones, with data acquisition performed at the center of each zone to establish localized response patterns, as shown in Figure 2. Each test was repeated 10 times to ensure consistency, generating 3240 damage signals and 360 baseline (healthy) signals in total. A representative LW signals for three damage severities and nine locations are shown in Figure 2. This setup allowed for robust and comprehensive data collection, covering multiple damage severities and locations across the CFRP laminate. The collected data set provides a strong foundation for DL-based damage detection, severity assessment, and localization.

### 2.2. Data Pre-Processing

#### 2.2.1. Data Normalization

Data normalization is an important pre-processing step in developing DL models, especially for LW signal analysis in SHM applications. Normalizing data scales the values to a specific range, which reduces variability and ensures consistent feature ranges across the dataset. This process improves model convergence during training by preventing features with larger magnitudes from disproportionately influencing the learning process, ultimately enhancing model accuracy and robustness. In this study, data normalization was implemented using a Min–Max scaler, which linearly transforms the raw LW signal data to a standardized range of [−1, 1]. This normalized range allows each feature to contribute equally, minimizing the effects of outliers and differing signal intensities. Such scaling is particularly advantageous in DL, as it leads to more efficient feature extraction by standardizing input data, without altering the inherent signal properties.

Following normalization, the dataset was divided into the training and testing sets in a 60:40 ratio. The training set, which included 60% of the normalized data, was subsequently increased via data augmentation to improve the generalization capabilities of the DL model, as discussed in the next section. The remaining 40% of the testing set was kept as unseen data to assess the built DL models. This partitioning strategy provides a solid framework to validate the ability of the model to precisely detect, localize, and assess damage severity in composite structures based on raw LW signals.

#### 2.2.2. Data Augmentation

Data augmentation is known to improve the performance of DL models, particularly with limited datasets [34,38]. By introducing controlled variations, augmentation significantly expands the training set and improves the model’s ability to generalize, which is essential for robust performance in real-world conditions. In this study, zero-mean Gaussian noise was added to the raw LW signals in the training set with standard deviations of 0.025 and 0.05. This approach tripled the training dataset, making the model more resilient to noise, and capable of handling the signal variability commonly encountered in SHM applications. Augmentation with zero mean Gaussian noise also prepares the DL model to accurately interpret signals, even when affected by environmental factors or minor sensor inconsistencies [39]. The augmented training data was then divided into 80% training and 20% validation datasets. This split allowed the model to train on a comprehensive dataset while using a dedicated validation set to assess its performance during development. By exposing the model to both noise-augmented training data and distinct validation data, this approach enabled precise model tuning, enhancing its reliability in detecting, localizing, and accurately assessing damage severity, even under varied and noisy conditions.

### 2.3. Deep Learning

SHM has evolved significantly with advancements in AI-based methods, especially DL. DL methods are of interest in damage identification and structural condition assessment due to their ability to autonomously extract damage-sensitive features, unlike traditional machine learning methods that require manual feature extraction. This study further explores the use of raw LW data with DL to directly capture the underlying features essential for damage detection, severity assessment, and localization. This approach simplifies the process by eliminating the need for extensive data pre-processing. In contrast, imaging-based methods require additional computational resources for pre-processing and post-processing, which increase complexity and reduce efficiency in practical applications. Therefore, DL has attracted significant attention in SHM, particularly concerning damage identification and the assessment of structural states. Among various DL models, ANN, CNN, and GRU have shown great promise in addressing SHM challenges for various types of structures [18,20,28]. Therefore, this study explores the potential of these three DL models. However, previous studies focused mostly on damage detection and the use of imaging-based methods; thus, this study proposes the use of raw LW data for damage detection, severity assessment, and localization.

#### 2.3.1. Artificial Neural Networks

**Figure 3:**
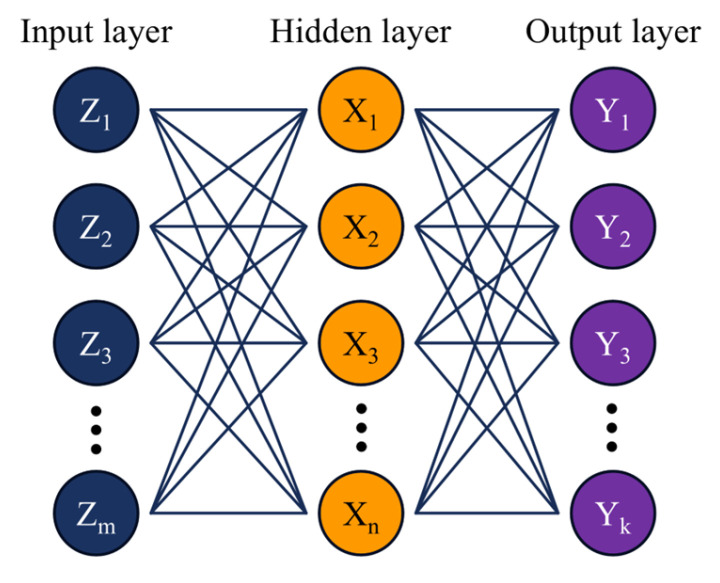
A typical ANN model with an input layer, a single hidden layer, and an output layer.

ANNs are intelligent computational algorithms that are designed to emulate the learning mechanisms of the human brain [40]. They consist of inter-connected artificial neurons in the form of layers, enabling the network to learn complex relationships and patterns within data by adjusting weights through iterative training [41]. A typical ANN model consists of at minimum one hidden layer sandwiched between an input and an output layer. Figure 3 shows an ANN model comprising one hidden layer, where:

(1) $$Input\;vector=\vec{Z}=\left(Z_{1},Z_{2},Z_{3},\ldots ,Z_{m}\right)$$

(2) $$Hidden\;layer\;vector=\vec{X}=\left(X_{1},X_{2},X_{3},\ldots ,X_{n}\right)$$

(3) $$Output\;vector=\vec{Y}=\left(Y_{1},Y_{2},Y_{3},\ldots ,Y_{n}\right)$$

Herein, m, n, and k are the number of neural units in the input, hidden, and output layers, respectively. Based on the series of inputs through the input neurons, the neural units in the hidden and output layers can be described as:

(4) $$x_{n}=g_{1}\left(\sum_{m=1}^{M}v_{nm}.z_{m}+b_{m}^{x}\right)$$

(5) $$y_{k}=g_{2}\left(\sum_{n=1}^{N}w_{kn}.x_{n}+b_{n}^{y}\right)$$

where, g represents the activation function that introduces non-linearity, v and w are the weight parameters that connect the input with the outputs, z and x represent the input to the hidden and output layer, respectively, b represents the bias terms, while M and N denote the numbers of neural units in the output layer.

#### 2.3.2. Convolutional Neural Network

**Figure 4:**
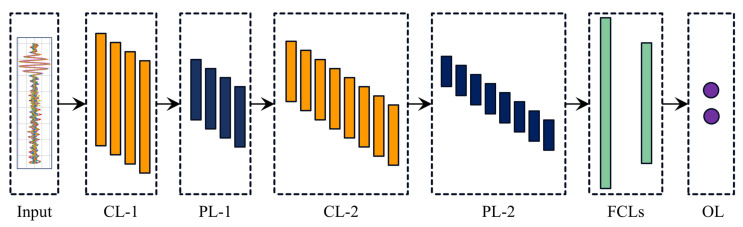
A typical CNN model comprises two convolutional layers (CLs), two pooling layers (PLs), two fully connected layers (FCLs), and an output layer (OL).

CNNs are inspired by the configuration of the mammalian visual cortex and are commonly applied to regression and classification tasks, owing to their capability to autonomously extract and learn hierarchical spatial features from input data [42]. A typical CNN architecture involves three important layer types: convolutional layers, pooling layers, and fully connected layers (FCLs), together with the final classification or regression layer [43]. Figure 4 shows a 1D−CNN model consisting of two convolutional and two FCL layers. The implementation of the convolutional operation at an input signal to extract relevant features is described as:

(6) $$y_{k}^{l}=f\left(b_{k}^{l}\sum_{i=1}^{N_{l-1}}Conv1D(w_{ik}^{l-1},s_{i}^{l-1})\right)$$

where, y represents the output features extracted by implementing convolutional operation at layer l, f is the activation function that introduces non-linearity to the output layer, b represents the bias term with the k-th output in layer l, N represents the number of features/channels taken as input from the previous layer, Conv1D represent the convolutional operation implemented on a 1D time series LW signal, w is the weight associated with the convolutional kernel, and s represents the input feature map from the preceding layer. In a typical CNN model, a pooling layer follows a convolutional layer to perform a down-sampling option for dimensionality reduction. After several convolutional and pooling layers, there are two FCLs, where each neural unit is linked to its counterpart in the preceding layer, allowing it to combine features learned by earlier layers for final classification or regression tasks. The final output layer consists of several neurons that are equivalent in number to the number of outputs required by the classification or regression tasks.

#### 2.3.3. Gated Recurrent Unit

**Figure 5:**
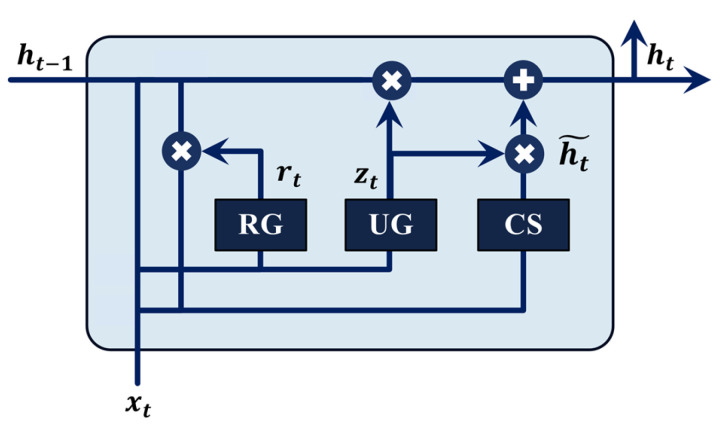
A typical cell of the GRU model comprises a reset gate (RG), an update gate (UG), and a candidate state (CS).

GRUs are a sub-class of recurrent neural networks (RNNs) that are capable of handling sequential data by efficiently capturing long-range dependencies across time steps. This feature of the GRU makes it well-suited for tasks involving time series analysis [44]. GRUs achieve this by employing a gating mechanism that dynamically controls the flow of information through the network, ensuring efficient handling of both short-term and long-term dependencies. The gating mechanism containing two main gates, an update gate, and a reset gate [28,45], controls the influence of past hidden states and current inputs on the computation of new hidden states. The update gate determines how much of the past information is retained, facilitating the preservation of long-term dependencies, while the reset gate decides how much past information is ignored, enabling the network to focus on relevant recent inputs. Together, these gates dynamically balance the contribution of past and current information, ensuring effective sequential data processing. Figure 5 shows a typical GRU cell with basic gates and states. The update gate identifies how much of the past information should be carried forward to future steps, while the reset gate controls the degree of influence of the previous hidden state on the current computation, and allows the network to disregard the unnecessary information from the past. For a given time, step t, and hidden state, $h_{t}$, the update gate is described as:

(7) $$z_{t}=\sigma \left(W_{z}.\left[h_{t-1},x_{t}\right]+b_{z}\right)$$

where, $z_{t}$ denotes the update gate output, $W_{z}$ denotes the weight matrix for the update gate, $h_{t-1}$ denotes the preceding hidden state, $x_{t}$ denotes the current input, and $b_{z}$ is the bias term. Thus, the amount of the previous hidden state retained in the current state is controlled by the update gate, which determines its contribution to the current state. Similarly, the reset gate is described as:

(8) $$r_{t}=\sigma \left(W_{r}.\left[h_{t-1},x_{t}\right]+b_{r}\right)$$

where, $r_{t}$ denotes the reset gate output, $W_{r}$ denotes the reset gate’s weight matrix, and $b_{r}$ denotes the bias term. The candidate hidden state introduces new information, regulated by the reset gate, enabling selective integration of past data. The final hidden state at time *t* merges this candidate state ($\tilde{h_{t}}$) with the previous hidden state, weighted by the update gate to effectively balance old and new information. This enables the GRU to efficiently capture sequential information, making it suitable for processing temporal raw LW signals in the SHM of composite structures.

## 3. Results and Discussion

### 3.1. Damage Detection and Severity Assessment

**Figure 6:**
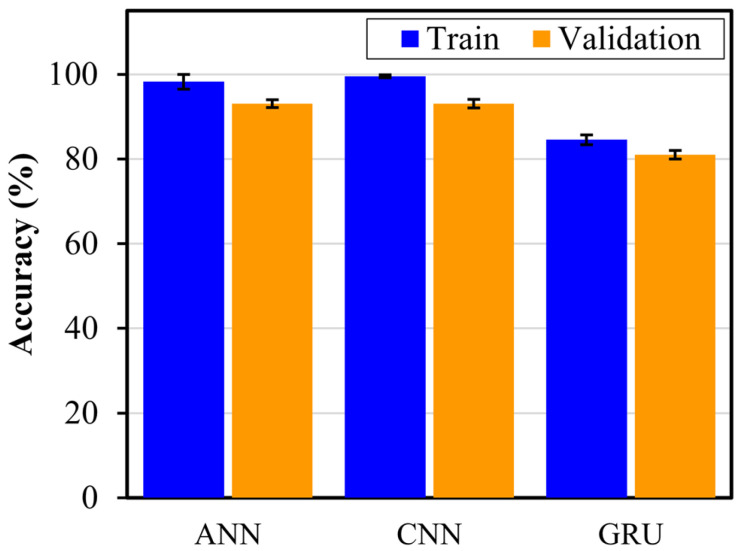
The training and validation accuracy for the trained DL models.

The deep learning models (ANN, CNN, and GRU) were trained using the prepared training and validation datasets to achieve accurate damage detection and severity assessment. Each DL model was trained for 50 epochs using the categorical cross-entropy loss function. The training process employed the Adam optimizer with a fixed learning rate of 0.001. These training parameters were selected to optimize convergence and ensure consistent learning across all DL models. To further improve the DL models, the architecture of all DL models was optimized through a random search technique, which systematically explored the number of layers and number of neurons to identify the most effective layer configurations. To further ensure the reliability and robustness of the model evaluations, a 5-fold cross-validation technique was applied. Through this approach, the data was split into five subsets, with each subset taking a turn as the validation set, while the others were used for training. By repeating this process five-fold, each model’s performance was comprehensively evaluated, reducing the likelihood of overfitting, and providing a more accurate measure of generalization capability. Figure 6 presents the training and validation accuracies for each model, illustrating the performance of each model after 50 epochs. The close alignment between the training and validation accuracy across all models indicates minimal overfitting, with the models maintaining high training and validation accuracies. The CNN-based DL model showed the best training accuracy of 99.57%, followed by ANN and GRU at 98.26% and 84.57%, respectively. However, in terms of validation accuracy, both ANN and CNN showed the same validation accuracies of 93.10%, while the GRU model showed a relatively lower validation accuracy of 81.03%. The high training and validation accuracies achieved by the CNN and ANN models suggest their ability to capture the spatial features and changes in signal patterns associated with different damage severities, which are essential for accurate detection and assessment. The GRU model, while lower in accuracy, still captures relevant temporal patterns in the LW signals, reflecting its strength in handling temporal data.

**Figure 7:**
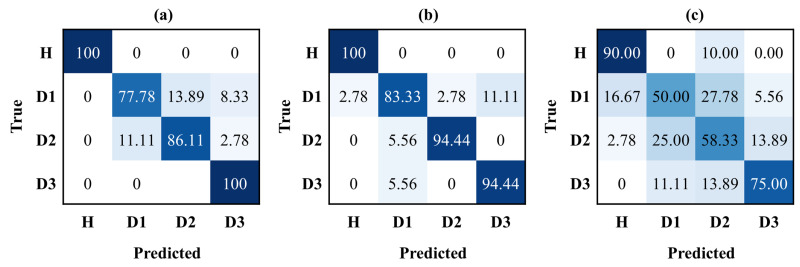
The confusion matrix represents the correct and incorrect prediction for damage detection and severity assessment on unseen test data for (**a**) ANN, (**b**) CNN, and (**c**) GRU.

**Figure 8:**
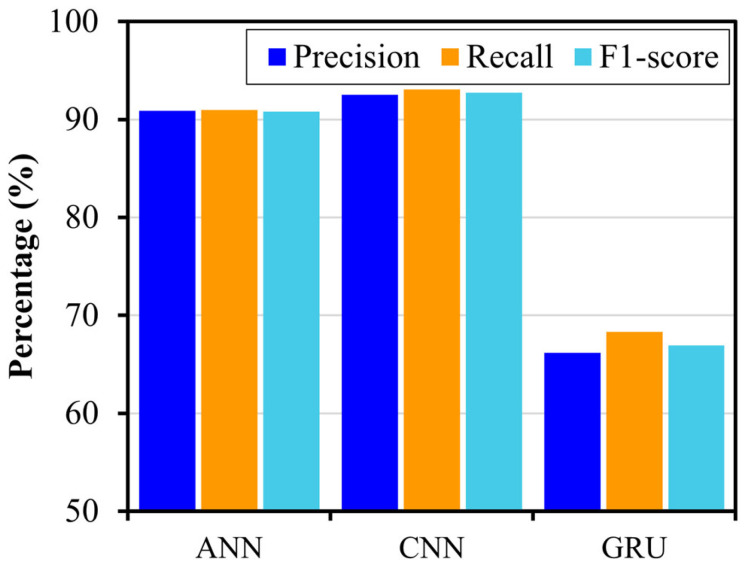
The precision, recall, and F1-scores for the ANN, CNN, and GRU models on the test set.

The trained DL models were further evaluated on unseen test data to assess their generalization capabilities. The testing accuracy for ANN, CNN, and GRU was 89.84%, 92.19%, and 65.62%, respectively, with CNN showing the highest performance, and GRU showing the worst performance, indicating over-fitting. Figure 7 shows the results of test data in terms of the confusion matrix. The confusion matrix demonstrates that ANN and CNN achieved 100% accuracy in identifying the healthy state, while GRU achieved 90%. When detecting damaged states regardless of severity, the combined accuracy for ANN, CNN, and GRU was 87.96%, 90.74%, and 61.11%, respectively, indicating that ANN and CNN are reliable for damage detection, with CNN performing slightly better overall. In damage severity assessment, the results showed that the higher severities (D2 and D3) were well captured by LWs, allowing the DL models to effectively learn features linked to severe damage, whereas lower severity damage (D1) presented more classification challenges across all models. Overall, the CNN model performed better in severity assessment for D1, D2, and D3, with accuracies of 83.33%, 94.44%, and 94.44%, respectively, demonstrating its superior ability to capture detailed features related to different levels of damage. Figure 8 shows the precision, recall, and F1-scores, which further highlight the capabilities of the DL models in damage detection and severity assessment. The CNN model revealed the highest scores with precision, recall, and F1-score of 92.52%, 93.06%, and 92.74%, respectively, reflecting its effectiveness in capturing the spatial features in LW data necessary for differentiating damage levels. The ANN model also showed slightly lower performance compared to the CNN model, indicating that the convolutional operations can help extract the discriminative features more efficiently from LW, as compared to the dense layers only. The GRU model, with precision, recall, and F1-score of 66.18%, 68.33%, and 66.91%, respectively, showed the lowest performance, likely due to its focus on temporal patterns, rather than spatial features. Thus, although the GRU can capture long-range dependencies, it is not able to effectively extract sensitive features for damage severity from the LW data. Additionally, the GRU model relies more on sequential processing, which limits its efficiency in identifying the spatial relationships among the provided sensing paths, which are critical for damage quantification. In contrast, the ANN model shows better feature extraction ability compared to GRU, due to its focus on extracting spatial relationships between sensing paths, instead of sequential order. Moreover, the CNN model provides a more advanced ability to extract localized spatial features using convolutional operations, making it particularly effective in identifying subtle changes in signals with different levels of severity and capturing the spatial dependencies that are critical for accurate damage detection and severity assessment. Therefore, the 1D−CNN model outperformed both ANN and GRU in terms of accuracy, precision, recall, and F1-score, demonstrating a superior capability for damage detection and severity assessment. Table 1 presents the architectural details of the proposed 1D CNN-based DL model.

### 3.2. Damage Localization

**Figure 9:**
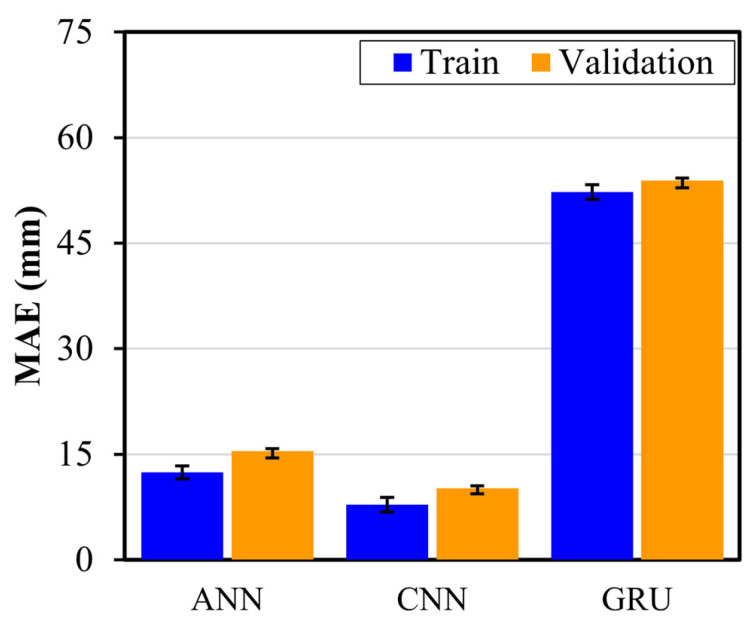
The training and validation MAEs for the trained DL models.

The DL models developed in Section 3.1 for damage detection and severity assessment were adapted for damage localization by replacing the final classification layer with a regression layer to predict the damage location in *x* and *y* coordinates. Localization performance was evaluated using the mean absolute error (MAE) and coefficient of determination (R^2^) to evaluate the ability of the model to accurately identify damage locations. Figure 9 shows the MAEs for ANN, CNN, and GRU. In training and validation, the CNN model achieved the best overall localization performance, with a training MAE of 7.81 mm and R^2^ of 97.37%, and a validation MAE of 10.17 mm and R^2^ of 95.78%, indicating effective spatial feature extraction. The ANN model showed reasonable localization capability with a training MAE of 12.44 mm and R^2^ of 90.76%, and a validation MAE of 15.46 mm and R^2^ of 89.34%. In contrast, the GRU model exhibited poor localization results, with training and validation MAE of 52.28 mm and 53.89 mm, respectively.

**Figure 10:**
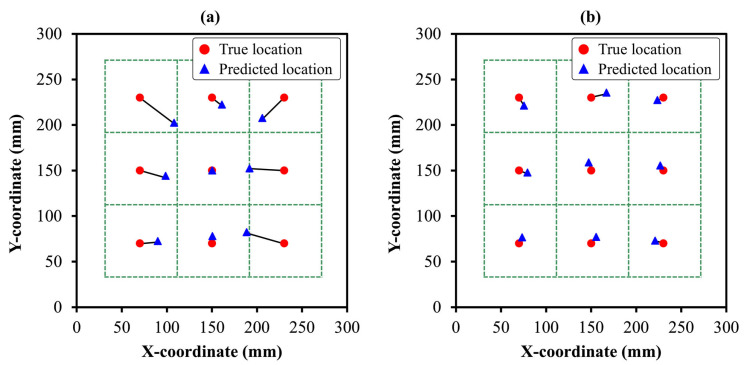
The average predicted damage locations by the DL model showing effective performance using (**a**) ANN, and (**b**) CNN.

Similar to the damage detection and severity assessment model, the damage localization model was also evaluated on an unseen test dataset. On the unseen test set, the 1D−CNN model again outperformed the other DL models with an x-coordinate MAE of 13.32 mm and R^2^ of 93.44%, and a y-coordinate MAE of 11.07 mm and R^2^ of 94.19%, resulting in an overall MAE of 12.20 mm and R^2^ of 93.82%. This strong performance demonstrates the robustness of the CNN model in effectively capturing the spatial features required for precise damage localization. The ANN model displayed moderate test performance, with an *x*-axis MAE of 24.07 mm and R^2^ of 81.33%, and a *y*-axis MAE of 13.84 mm with R^2^ of 91.33%, yielding an overall MAE of 18.95 mm and R^2^ of 86.33%, indicating adequate but less precise localization, compared to CNN. The GRU model with an overall MAE of 54.45 mm was unable to localize the damage well, demonstrating its limitation in spatial localization. Thus, similar to damage detection and severity assessment, the GRU model is unable to identify the LW patterns for damage location, due to its focus on sequential information. However, the ANN and CNN models showed better feature extraction ability for damage localization as well. Figure 10 presents the average predicted damage locations by the better damage localization models (ANN and CNN), alongside the true damage locations in the composite structure. The CNN model (Figure 10b) exhibits superior localization accuracy, with predicted points closely aligning with true damage coordinates, demonstrating its capability to effectively capture spatial dependencies within LW data. Conversely, the ANN model (Figure 10a) displays slightly larger deviations from the true locations, particularly when the locations are far from the origin, indicating comparatively reduced spatial precision. Moreover, the results highlight the effectiveness of processing raw LW signals using CNN and prove its advantage over imaging methods by showing low MAE. This signifies the direct capturing of wave propagation features through CNN by eliminating complex pre-processing steps and additional computation requirements of imaging methods. These observations highlight the precision of the CNN model to accurately localize damage, demonstrating its advantage in handling both damage severity assessment and localization in the SHM of laminated composites.

## 4. Conclusions

This study developed a DL-based framework for the simultaneous damage severity assessment and localization of laminated composite structures using raw LWs. Three DL models, namely ANN, CNN, and GRU, were explored for this purpose. For damage severity assessment, CNN showed a test accuracy of 92.19% and an F1 score of 92.74%, showcasing its effectiveness in distinguishing between varying levels of structural damage. In terms of damage localization, the CNN model also showed better performance, achieving a test MAE of 12.20 mm, showcasing its precision in locating the damage. In comparison, the ANN model displayed moderate localization capability with an MAE of 18.95 mm, while the GRU model was less effective with a significantly higher MAE of 54.45 mm, underscoring its limitations in spatial feature extraction. The superior accuracy and localization precision of the CNN model demonstrates its potential for application in SHM, providing a scalable and reliable solution for predictive maintenance using raw LW signals, rather than computationally expensive imaging methods, across fields such as aerospace and civil engineering, where maintaining structural integrity is critical. Despite the promising results of the CNN-based DL model, this work has focused on damage severity assessment and localization for laminated composite plates using raw LW signals. Therefore, future work could extend the proposed approach to complex composite structures, such as stiffened panels and sandwich structures, and the application of hybrid DL methods for improved performance. Additionally, the proposed approach could also be extended to real-time monitoring of composite structures in future studies.

## Figures and Tables

**Table 1:** The architectural details of the 1D−CNN-based optimum model for damage severity assessment and localization.

Layer	Output Shape	Parameters	Optimized Hyperparameters
Input	(None, 1500, 12)	-	-
Conv1D	(None, 1498, 16)	592	Filters: 16, Kernel Size: 3, Activation: ReLU
MaxPooling1D	(None, 749, 16)	-	Pool Size: 2
Conv1D	(None, 747, 32)	1568	Filters: 32, Kernel Size: 3, Activation: ReLU
MaxPooling1D	(None, 373, 32)	-	Pool Size: 2
Conv1D	(None, 371, 64)	6208	Filters: 64, Kernel Size: 3, Activation: ReLU
MaxPooling1D	(None, 185, 64)	-	Pool Size: 2
Flatten	(None, 11840)	-	-
Dense	(None, 64)	757,824	Units: 64, Activation: ReLU
Dense (Severity)	(None, 4)	260	Activation: Sigmoid
Dense (Localization)	(None, 2)	130	Activation: Linear

## Data Availability

The data presented in this study are available on request from the corresponding author.
